# The association between ADHD and physical health: a co-twin control study

**DOI:** 10.1038/s41598-020-78627-1

**Published:** 2020-12-28

**Authors:** Pei-Yin Pan, Sven Bölte

**Affiliations:** 1 Center of Neurodevelopmental Disorders (KIND), Centre for Psychiatry Research, Karolinska Institutet & Stockholm Health Care Services, Region Stockholm, Stockholm, Sweden; 2 Department of Women’s and Children’s Health, Karolinska Institutet & Stockholm Health Care Services, Solna, Sweden; 3 Child and Adolescent Psychiatry, Karolinska Institutet, Stockholm, Sweden; 4 Curtin Autism Research Group, School of Occupational Therapy, Social Work and Speech Pathology, Curtin University, Perth, WA Australia

**Keywords:** Diseases, Medical research, Risk factors

## Abstract

Attention-deficit/hyperactivity disorder (ADHD) has been associated with increased risk for physical comorbidity. This study used a twin cohort to investigate the association between physical diseases and phenotypic variations of ADHD. A twin cohort enriched for ADHD and other neurodevelopmental conditions were analysed. The Attention Problems subscale of the Child Behavior Checklist/Adult Behavior Checklist (CBCL/ABCL-AP) was used to measure the participants’ severity of ADHD symptoms. Physical health issues were obtained with a validated questionnaire and were tested in relation to ADHD symptom severity in a co-twin control model. Neurological problems were significantly associated with a diagnosis of ADHD. A conditional model for the analysis of within-twin pair effects revealed an inverse association between digestive problems and the severity of ADHD symptoms, after adjusting for co-existing autism spectrum disorder and ADHD medications. Our findings suggest that individuals with ADHD are susceptible to neurological problems, why a thorough neurological check-up is indicated in clinical practice for this population. In addition, health conditions of digestive system could be considered as a non-shared environmental factor for behavioral phenotypes in ADHD. It supports the possible role of gut-brain axis in the underpinnings of ADHD symptoms, at least for a subgroup of individuals with certain genetic predisposition.

## Introduction

Attention-deficit/hyperactivity disorder (ADHD) is a common neurodevelopmental condition, defined by impairing symptoms of inattention, impulsivity, and hyperactivity^1^. ADHD is a complex heritable condition and evidence indicates that the physiology underlying ADHD involves alterations of brain monoaminergic neurotransmitter systems^2^ and reduced connectivity of brain neural networks^3^ leading to behavioural phenotypes characterized by a range of cognitive challenges in executive functioning and reward processing. However, while an interplay of polygenic liability and environmental factors during crucial time windows of brain development is assumed, there is still insufficient evidence to support clear causal pathways to ADHD^4^. Environmental factors of ADHD have received considerable research interest in spite of the high heritability of the condition^4^. Recent studies indicate that environmental risks might not only account for up to 40% of the variability of ADHD symptomatology^5^, but could also contribute largely to its heritability through gene-environment interactions or correlations^4^. Among the potential environmental exposures, the effect of health conditions in physical systems on the variation of ADHD symptoms has not yet been thoroughly investigated, albeit for instance, dietary interventions having shown some positive influence on core symptomatology in children with ADHD via digestive system^6^.

There have been several physical health conditions reported to be associated with ADHD. For example, it was found that children with ADHD are at 2.5-fold risk to develop unprovoked seizure^7^. Childhood seizure also appear to increase the risk for ADHD up to five times compared to those without seizures^8^. In addition, an association between migraine and ADHD has been supported by a recent meta-analysis of epidemiological studies^9^. Other common physical comorbidities among the ADHD population are immunological dysregulation, including asthma, allergic rhinitis, and atopic eczema^10,11^, obesity and overweight^12^, as well as altered gut microbiome functions^13^. The higher rates of comorbid ADHD and physical health issues might imply the possibility of shared genetic susceptibility and/or environmental adversities which affect multiple systems increasing the likelihood of the emergence of overlaps between ADHD and physical illness. The probability that biological pathways of a specific physical problem are partly involved in the etiological mechanisms of ADHD may increase, if ADHD phenotypes covary with the presence of physical illness. Based on these assumptions, individuals with ADHD and co-occurring physical conditions could be considered to qualify as stratification subgroups with specific etiological pathways involved, where targeted biological intervention might be meaningful and clinically feasible^14^.

The current knowledge on the relationship between physical comorbidity and the severity of ADHD symptoms is still scarce, and the results of previous studies are inconsistent. For example, although studies on epilepsy in ADHD reported that children with higher seizure frequency and poorer seizure control may show increased levels of inattention and hyperactivity symptoms^15−17^, no age of onset, type of epilepsy, or interictal EEG change seems predictive of ADHD or its symptoms^18^. In addition, despite the potential role of neuroinflammation in ADHD etiology, interventions for immune dysregulation in ADHD still lack evidence^19^. Another physical system which has more recently been implicated in ADHD is the gastrointestinal (GI) tract, which communicates with brain bidirectionally via the gut-brain axis in which nervous, endocrine, and immunological pathways are involved^20^. One reason is the association between gut microbiota composition and behaviour in animal models, including motor activity^21^, and another are potentially promising, albeit still rather emerging and experimental, dietary interventions for ADHD^6^. While a couple of studies showed that individuals with ADHD were more likely to exhibit GI problems compared to those without^22,23^, it remains unclear whether there are characteristics of GI tract health conditions which could be candidate biomarkers to identify those who are at risk for ADHD or the potential responders to dietary interventions.

The nature of ADHD genetics might complicate the elucidation of environmental contributions to the condition, and its variable phenotypes, such as the role of co-occurring physical conditions. First, a combination of common variants mainly constitutes ADHD etiology indicating clinical diagnosis of ADHD is an extreme expression of continuous heritable traits^4,24^. Second, in addition to genetic stability, evidence supports that genetic innovations are also associated with ADHD symptoms throughout the brain development^25^. Hence, studies investigating monozygotic twins discordant for ADHD diagnosis or dimensional symptom variations are particularly powerful to unravel the role of environmental factors in the emergence of ADHD symptoms^26,27^. Twin studies enable maximal control of genetic confounding and other bias, such as age, gender, shared environment, and early family experiences. Thus, findings from such study designs are more informative to support causal inferences that are not biased by uncontrolled confounders.

The objective of the present study was to apply twin design to contrast twins differing in the severity of ADHD symptoms to disentangle the role of comorbid physical health problems for the underpinnings of ADHD phenotypes across the full range of inattentive and hyperactive-impulsive behavior manifestations. So far, to the best of our knowledge, this is the first study to address physical health in ADHD using this informative approach. We sought to analyze a twin cohort enriched for ADHD, autism spectrum disorder (ASD), and other neurodevelopmental disorders (NDDs) to explore the distribution of physical health conditions in twin pairs who are qualitatively (for ADHD diagnosis) discordant or quantitatively (for dimensional ADHD symptoms) differing for ADHD phenotypes. Additionally, we examined the association between physical health and the severity of ADHD symptoms using a co-twin control design to account for genetic influence and other possible shared confounds on their relation. Since ASD and intellectual disability are themselves often associated with physical problems^28,29^, we included ASD diagnosis and IQ as covariates in our analyses.

## Methods

### Participants

**Figure 1:**
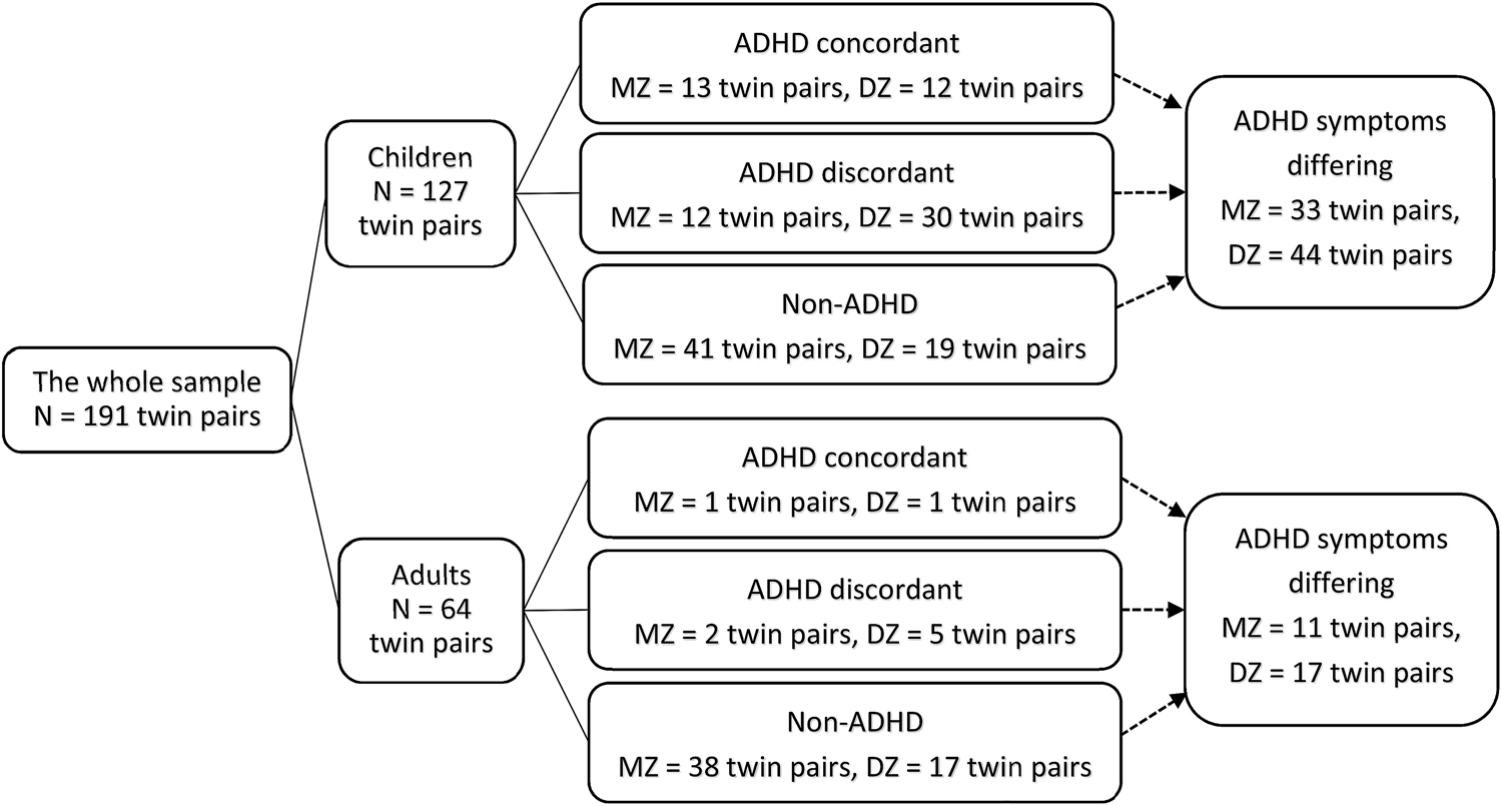
Twin pairs in analyses of the association between somatic comorbidity and clinical (ADHD) and quantitative (ADHD symptoms) ADHD phenotypes. *ADHD* attention-deficit/hyperactivity disorder, *MZ* monozygotic twins, *DZ* dizygotic twins.

Sample characteristics and composition regarding ADHD concordance within twin pairs are presented in Fig. 1 and Table 1. Twins included in this study were recruited between August 2011 to June 2019 within the Roots of Autism and ADHD Twin Study Sweden (RATSS)^30^. Participants in RATSS are referred from the Child and Adolescent Twin Study in Sweden (CATSS), (a) Swedish nationwide population-based twin study focusing on children’s somatic and mental health since 1994^31^ (42.0%); (b) the National Swedish Patient Registry by the Swedish Board of Health and Welfare, (c) clinical departments in Region Stockholm (Child and Adolescent Psychiatry, Rehabilitation and Health centers, and (neuro-) pediatric units); (d) summons in social and print media by national interest organizations for neurodevelopmental conditions, and twin organizations. RATSS enrols twin participants who are discordant or concordant for ASD, ADHD, and other NDDs, as well as concordant typically developing (TD) twin controls. In the present study, 382 twins from 191 pairs were included in the analysis, 107 monozygotic (MZ) and 84 dizygotic (DZ) pairs (mean age = 16.62 ± 5.92 years, range 8–33). Children (< 18 years) accounted for the majority of pairs (127 pairs, 66.6%). Zygosity was determined by standard methods of DNA testing with saliva or whole-blood samples as described previously^32,33^. Within our sample, there were 49 twin pairs discordant for a clinical diagnosis of ADHD (14 MZ pairs and 35 DZ pairs), and 27 ADHD concordant twin pairs (14 MZ pairs and 13 DZ pairs). Additionally, 105 twin pairs differed dimensionally for ADHD symptoms (44 MZ pairs and 61 DZ pairs), as defined by an intra-pair difference on the score of the Attention Problems subscale in the Child Behavior Checklist/Adult Behavior Checklist (CBCL/ABCL-AP) of at least 2 points, corresponding to 1 standard error of measurement in our sample (1.70 for CBCL-AP, 1.84 for ABCL-AP, respectively). With this definition, the mean scores of CBCL-AP for dimensionally differing twins were 9.44 (twins with higher scores) and 3.74 (the co-twin with lower scores), and the mean scores of ABCL-AP were 8.93 (twins with higher scores) and 2.79 (the co-twin with lower scores). The distributions of CBCL/ABCL-AP scores in groups with different age strata and zygosity are displayed in Table 1.

**Table 1:** Sample characteristics.

	Total sample (all ages/children/adults)		Sample for within-pair difference analyses							
			Twins discordant for ADHD diagnosis (all ages/children/adults)				Twins differing for ADHD symptoms (all ages/children/adults)			
			MZ		DZ		MZ		DZ	
Pairs (n)	191/127/64		14/12/2		35/30/5		44/33/11		61/44/17	
**Age**										
Mean	16.62/13.11/23.59		14.14/12.33/25.00		13.51/12.47/19.80		16.02/13.48/23.64		15.33/12.73/22.06	
SD	5.92/2.55/4.29		5.16/2.41/3.46		3.44/2.21/2.78		5.41/4.82/3.98		5.01/2.37/3.56	
Range	8–33/8–17/18–33		9–28/9–17/22–28		8–25/8–16/18–25		8–28/8–17/18–28		8–31/8–17/18–31	

	ADHD	Non-ADHD	ADHD twin	Co-twin	ADHD twin	Co-twin	Higher attention score	Lower attention score	Higher attention score	Lower attention score
Subjects (n)	103/92/11	279/162/117	14/12/2	14/12/2	35/30/5	35/30/5	44/33/11	44/33/11	61/44/17	61/44/17
**Sex, N subjects**										
Male	64/61/3	137/89/48	16/16/0		22/19/3	21/18/3	56/50/6		32/23/9	37/27/10
Female	39/31/8	142/73/69	12/8/4		13/11/2	14/12/2	32/16/16		29/21/8	24/17/7
**NDD diagnosis, N subjects**										
ADHD	103/92/11	0	14/12 /2	0	35/30/5	0	20/18/2	11/11/0	35/32/3	10/8/2
ASD	41/37/4	47/33/14	6/5/1	1/1/0	14/13/1	7/6/1	20/17/3	11/10/1	22/14/8	10/9/1
ID	10/10/0	10/7/3	2/2/0	0/0/0	1/1/0	0/0/0	7/7/0	3/3/0	2/0/2	2/2/0
Other NDDs	31/28/3	33/22/11	3/3/0	1/1/0	8/7/1	8/5/3	9/9/0	6/5/1	15/12/3	8/5/3
**Full-scale IQ**										
Mean	94.42/94.37/94.82	100.83/100.49/101.30	99.15/98.27/104.00	100.86/100.67/102.00	97.74/97.72/97.80	100.29/101.60/92.40	90.19/86.90/99.45	94.95/92.34/102.55	97.38/98.61/94.00	102.77/104.68/97.82
SD	17.08/17.59/12.84	14.93/13.95/16.25	20.60/20.78/26.87	10.84/11.58/7.07	15.59/16.49/10.09	10.63/10.77/5.41	17.29/16.55/16.60	15.47/14.35/16.80	15.47/15.06/16.55	14.59/14.86/12.99
**Attention subscale score in CBCL/ABCL**										
Mean	NA/10.05/11.29	NA/3.11/3.73	NA/8.78/11.00	NA/4.11/6.00	NA/10.57/10.00	NA/2.39/10.67	NA/9.36/6.36	NA/5.15/3.00	NA/9.50/10.59	NA/2.68/2.65
SD	NA/3.87/3.59	NA/3.42/4.60	NA/2.95/2.83	NA/2.32/2.83	NA/3.99/2.65	NA/2.85/12.66	NA/3.63/4.11	NA/4.00/3.63	NA/4.22/5.96	NA/3.18/3.43

*ASD* autism spectrum disorder, *ADHD* attention-deficit/hyperactivity disorder, *ID* intellectual disability, *NDD* neurodevelopmental disorder, *MZ* monozygotic twins, *DZ* dizygotic twins, *CBCL* the Child Behavior Checklist, *ABCL* Adult Behaviour Checklist.

### Ethical approval

All procedures performed in this study were approved by the Regional Swedish Ethical Review Board in Stockholm (ref: 2016/1452-31), and were in accordance with the 1964 Helsinki declaration and its later amendments or comparable ethical standards. Informed consents were obtained from all participants and/or their legal guardians.

### Diagnostic and behavioural assessments

Participating twins were evaluated using a comprehensive psychodiagnostic assessment administered by experienced clinicians in the RATSS team^30^. Clinical diagnoses were based on the Diagnostic and Statistical Manual of Mental Disorders, Fifth Edition (DSM-5)^1^, endorsed by results from standardized diagnostic instruments, including the Kiddie Schedule for Affective Disorders and Schizophrenia-Present and Lifetime Version (K-SADS-PL)^34^, and the Diagnostic Interview for ADHD in Adults (DIVA 2.0)^35^, the Autism Diagnostic Interview-Revised (ADI-R)^36^, and the Autism Diagnostic Observation Schedule Second Edition (ADOS-2)^37^. Full-scale IQ was estimated with the Wechsler Intelligence Scales for Children or Adults, Fourth Editions^38,39^. Dimensional ADHD symptoms were quantified with the CBCL/ABCL-AP, consisting of 10/17 items, assessing behaviour symptoms related to attention problems within the past 6 months. The CBCL (6- to 18-year-olds version)^40^ was completed by the parents, and the ABCL (for ages 18–59)^41^ was completed by the participant’s next of kin (mostly parents or spouse). Both the CBCL and ABCL are broadband screeners and part of the Achenbach System of Empirically Based Assessment (ASEBA), a family of tools evaluating problem behaviours and competencies from a wide range of perspectives, including internalizing and externalizing symptoms, as well as social problems and somatic complaints. Items on CBCL/ABCL are rated on a 3-point Likert scale (ranging from 0 to 2), with higher scores indicating more severe symptoms.

### Medical history and present physical comorbidity

A parent-/self-report questionnaire validated against medical registry data and designed to obtain information on medical history and present physical health issues is used in RATSS^42^. The questionnaire consists of one open question “Has the child (Have you) been seriously ill during his/her (your) childhood?” and 33 closed questions inquiring about whether the participant had ever had a specific physical health diagnosis or problem. The full list of physical conditions and the distribution of these physical health issues in our sample are summarized in Table 2, except for cardiovascular diseases, for which too few cases were reported to be included in the analysis (four in ADHD, seven in non-ADHD). The comorbid physical problems were categorized into groups based on different physical systems/etiologies (history of infectious diseases, neurological problems, gastrointestinal problems, and immune dysregulation). Different conditions in the same group were summed up to generate a predictive estimate (as a variable indicating the frequency of problems in each group) for clinical ADHD diagnosis or the severity of ADHD symptoms. The reasons to use this variable were: (1) our sample might not have enough power to detect the difference between ADHD and non-ADHD for each physical illness (2) we sought to examine the prognostic power of different levels of physical morbidity severity within each physical system on ADHD both categorically and dimensionally. Among these conditions, two of the items “migraine” and “headache” were combined into one condition “headache” and coded as one physical health issue since these two conditions were clinically highly correlated.

**Table 2:** Comparisons of physical problems between ADHD and non-ADHD.

(a) Comparison in the total sample (p value unadjusted)												
Physical systems	All ages				Children				Adults			
	ADHD	Non-ADHD	*t*	*p /H*_*1*_*:H*_*0*_	ADHD	Non-ADHD	*t*	*p /H*_*1*_*:H*_*0*_	ADHD	Non-ADHD	*t*	*p /H*_*1*_*:H*_*0*_
	n = 103	n = 279			n = 92	n = 162			n = 11	n = 117		
**Infectious diseases**												
Sum of diseases for each individual, mean	1.55	1.50	0.50	0.620/0.14	1.55	1.47	0.72	0.474/0.18	1.55	1.55	− 0.01	0.996/0.31
Measles, n (%)	0	5 (1.8)			0	5 (3.1)			0	0		
Mumps, n (%)	0	5 (1.8)			0	0			0	5 (4.3)		
Encephalitis, n (%)	1 (1.0)	2 (0.7)			1 (1.1)	1 (0.6)			0	1 (0.9)		
Chickenpox, n (%)	94 (91.3)	244 (87.5)			84 (91.3)	139 (85.8)			10 (90.9)	105 (89.7)		
Pertussis, n (%)	4 (3.9)	29 (10.4)			2 (2.2)	7 (4.3)			2 (18.2)	22 (18.8)		
Lyme disease, n (%)	3 (2.9)	12 (4.3)			3 (3.3)	8 (4.9)			0	4 (3.4)		
Scarlet fever, n (%)	9 (8.7)	14 (5.0)			9 (9.8)	10 (6.2)			0	4 (3.4)		
Frequent cold, n (%)	22 (21.4)	33 (11.8)			20 (21.7)	25 (15.4)			2 (18.2)	8 (6.8)		
Frequent ear infection, n (%)	23 (22.3)	62 (22.2)			20 (21.7)	35 (21.6)			3 (27.3)	27 (23.1)		
RS virus infection, n (%)	2 (1.9)	6 (2.2)			2 (2.2)	3 (1.9)			0	3 (2.6)		
Pneumonia, n (%)	2 (1.9)	7 (2.5)			2 (2.2)	5 (3.1)			0	2 (1.7)		
**Neurological problems**												
Sum of diseases for each individual, mean	0.62	0.38	**2.55**	0.012*/9.21	0.63	0.38	2.28	0.024/2.99	0.55	0.37	0.90	0.372/0.42
Epilepsy, n (%)	5 (4.9)	4 (1.4)			4 (4.3)	2 (1.2)			1 (9.1)	2 (1.7)		
Vertigo, n (%)	8 (7.8)	7 (2.5)			8 (8.7)	4 (2.5)			0	3 (2.6)		
Headache, n (%)	31 (30.1)	50 (17.9)			29 (31.5)	31 (19.1)			2 (18.2)	19 (16.2)		
Brain injury, n (%)	13 (12.6)	26 (9.3)			12 (13.0)	15 (9.3)			1 (9.1)	11 (9.4)		
Hearing impairment, n (%)	7 (6.8)	17 (6.1)			5 (5.4)	9 (5.6)						
**Gastrointestinal problems**												
Sum of diseases for each individual, mean	0.18	0.19	− 0.11	0.916/0.13	0.20	0.17	0.53	0.600/0.16	0.09	0.22	− 0.81	0.418/0.40
Lactose intolerance, n (%)	10 (9.7)	30 (10.8)			10 (10.9)	16 (9.9)			0	14 (12.0)		
Gluten intolerance, n (%)	1 (1.0)	3 (1.1)			1 (1.1)	2 (1.2)			0	1 (0.9)		
Irritable bowel, n (%)	1 (1.0)	5 (1.8)			0	1 (0.6)			1 (9.1)	4 (3.4)		
Diarrhea, n (%)	2 (1.9)	0			2 (2.2)	0			0	0		
Constipation, n (%)	1 (1.0)	4 (1.4)			1 (1.1)	4 (2.5)			0	0		
Stomach problems, n (%)	0	6 (2.2)			0	2 (1.2)			0	4 (3.4)		
Other GI problems, n (%)	4 (3.9)	5 (1.8)			4 (4.3)	2 (1.2)			0	3 (2.6)		
**Immunological problems**												
Sum of diseases for each individual, mean	0.78	0.68	0.94	0.348/0.19	0.77	0.74	0.26	0.793/0.15	0.82	0.60	0.83	0.410/0.40
Asthma, n (%)	21 (20.4)	54 (19.4)			19 (20.7)	38 (23.5)			2 (18.2)	16 (13.7)		
Eczema, n (%)	27 (26.2)	70 (25.1)			24 (26.1)	43 (26.5)			3 (27.3)	27 (23.1)		
Milk allergy, n (%)	5 (4.9)	12 (4.3)			5 (5.4)	7 (4.3)			0	5 (4.3)		
Allergy to specific allergen, n (%)	25 (24.3)	51 (18.3)			21 (22.8)	32 (19.8)			4 (36.4)	19 (16.2)		
Other immunological problems, n (%)	2 (1.9)	3 (1.1)			2 (2.2)	0			0	3 (2.6)		

(b) Comparison in twins discordant for ADHD diagnosis (p value unadjusted)																
Physical systems (sum of diseases for each individual, mean)	Monozygotic twins								Dizygotic twins							
	All ages, n = 14 pairs				Children, n = 12 pairs				All ages, n = 35 pairs				Children, n = 30 pairs			
	ADHD	Non-ADHD	Z	p/H_1_:H_0_	ADHD	Non-ADHD	Z	p/H_1_:H_0_	ADHD	Non-ADHD	Z	p/H_1_:H_0_	ADHD	Non-ADHD	Z	p/H_1_:H_0_
Infectious diseases	1.43	1.35	− 1.00	0.317/0.41	1.42	1.33	− 1.00	0.317/0.44	1.40	1.57	− 0.88	0.378/0.26	1.37	1.53	− 0.89	0.372/0.26
Neurological problems	0.64	0.28	− 0.88	0.830/0.43	0.67	0.33	− 0.65	0.516/0.39	0.43	0.37	− 0.42	0.672/0.20	0.43	0.43	− 0.04	0.971/0.19
Gastrointestinal problems	0.14	0.21	− 0.58	0.564/0.31	0.08	0.25	− 1.41	0.157/0.69	0.20	0.29	− 0.54	0.593/0.23	0.23	0.13	− 1.13	0.257/0.35
Immunological problems	1.00	1.07	− 0.38	0.705/0.29	0.92	1.08	− 0.82	0.414/0.38	0.51	0.86	− 1.40	0.161/0.52	0.53	0.87	− 1.32	0.186/0.46

(c) Comparison in twins differing for ADHD symptoms (p value unadjusted)																
Physical systems (sum of diseases for each individual, mean)	Monozygotic twins								Dizygotic twins							
	All ages, n = 44 pairs				Children, n = 33 pairs				All ages, n = 61 pairs				Children, n = 44 pairs			
	Higher attention score	Lower attention score	Z	p/H_1_:H_0_	Higher attention score	Lower attention score	Z	p/H_1_:H_0_	Higher attention score	Lower attention score	Z	p/H_1_:H_0_	Higher attention score	Lower attention score	Z	p/H_1_:H_0_
Infectious diseases	1.68	1.80	− 0.28	0.776/0.22	1.67	1.88	− 0.81	0.417/0.33	1.36	1.66	− 2.15	0.032/1.13	1.32	1.55	− 1.41	0.159/0.37
Neurological problems	0.59	0.34	− 1.46	0.145/0.53	0.73	0.45	− 1.19	0.236/0.42	0.43	0.51	− 0.53	0.596/0.17	0.50	0.50	− 0.16	0.870/0.16
Gastrointestinal problems	0.16	0.30	− 2.12	0.034/1.47	0.18	0.36	− 2.45	0.014/3.76	0.21	0.13	− 1.00	0.317/0.25	0.16	0.14	− 0.33	0.739/0.17
Immunological problems	1.00	1.16	− 1.24	0.216/0.36	0.97	1.12	− 0.91	0.361/0.29	0.57	0.72	− 0.93	0.352/0.22	0.57	0.82	− 1.26	0.209/0.36

*p < 0.0125.

With Bonferroni correction, the significance level of p values in this table is set at p < 0.0125. Brain injuries included hydrocephalus, cerebral palsy, intracerebral hemorrhage, congenital cerebral malformation, and concussion; other GI problems included gastroenteritis, intestinal polyps, abdominal pain, esophagitis, and biliary atresia; other immunological problems included type 1 diabetes, ankylosing spondylitis, and immunodeficiency.

*H*_*0*_ null hypothesis, *H*_*1*_ alternative hypothesis.

### Statistical analysis

All statistical analyses were performed with IBM SPSS software version 25 (SPSS Inc., Chicago, IL, USA), the drgee package^43^ and BayesFactor package^44^ in R version 3.5.1. Student’s t test was used to compare the frequency of physical comorbidity between the individuals with ADHD and without ADHD in the whole sample. To examine if the amount of physical problems differed within twins discordant for ADHD diagnosis or ADHD symptoms, Wilcoxon sign-rank test was used in consideration of the sample size of those twin pairs. Owing to the overall exploratory nature of the research approach, Bayes factors (alternative hypothesis [H_1_]: null hypothesis [H_0_]) were also calculated for these comparisons. For the co-occurring physical health conditions which were identified with significantly higher frequency in ADHD in the whole sample, the association with ADHD phenotypes (both categorical and dimensional) was tested. Conditional multivariate logistic and linear regression analysis with twin pairs clustered was used to explore the adjusted associations between physical health and ADHD diagnosis as well as dimensional ADHD symptoms, after adjustment for potential confounding variables. To determine the within pair effect of physical comorbidity on the variation of ADHD symptoms among quantitatively differing twins, we used conditional generalized estimating equations (CGEE), a multiply adjusted (conditional) linear regression model, to eliminate the influence of pair-consistent confounders^43^. Child (< 18 years) and adult (≥ 18 years) participants were analysed separately in addition to the whole sample which included participants of all ages, except for the adult discordant twins due to limited sample sizes. All tests were two-tailed and p-values of 0.05 or less were considered statistically significant. A Bonferroni correction was applied for multiple comparisons in all the analyses (p < 0.0125 in Tables 2 and 4; p < 0.05 in Table 3).

**Table 3:** Associations between neurological problems and ADHD in the total sample.

(a) ADHD diagnosis												
	Outcome: ADHD diagnosis											
	All ages (n = 382)				Children (n = 254)				Adults (n = 128)			
	β	s.e	p	OR (95% CI)	β	s.e	p	OR (95% CI)	β	s.e	p	OR (95% CI)
**Exposure variable**												
Neurological Problems	0.32	0.17	0.066	1.37 (0.98–1.92)	0.38	0.19	0.046*	**1.46 (1.01–2.12)**	0.01	0.33	0.981	1.01 (0.53–1.91)
**Covariate 1**												
Full-scale IQ	− 0.02	0.01	0.080		− 0.02	0.01	0.102		− 0.01	0.02	0.734	
**Covariate 2**												
Age	− 0.16	0.03	< 0.001		− 0.12	0.07	0.087		− 0.23	0.10	0.025	
**Covariate 3**												
Gender	0.36	0.28	0.195		0.63	0.30	0.037		− 1.19	0.80	0.137	
**Covariate 4**												
ASD	0.86	0.33	0.008		0.76	0.35	0.031		1.56	0.83	0.060	

(b) ADHD symptoms									
	Outcome: ADHD symptoms (CBCL/ABCL-attention subscale score)								
	All ages (n = 382)			Children (n = 254)			Adults (n = 128)		
	β	s.e	p	β	s.e	p	β	s.e	p
**Exposure variable**									
Neurological Problems	0.07	0.33	0.829	0.65	0.36	0.071	− 1.04	0.62	0.095
**Covariate 1**									
Full-scale IQ	− 0.09	0.02	< 0.001	− 0.08	0.02	< 0.001	− 0.07	0.02	0.003
**Covariate 2**									
Age	− 0.14	0.04	< 0.001	− 0.51	0.12	< 0.001	− 0.45	0.10	< 0.001
**Covariate 3**									
Gender	0.08	0.47	0.859	0.07	0.51	0.884	0.06	0.76	0.940
**Covariate 4**									
ASD	2.89	0.75	< 0.001	2.18	0.72	0.002	5.34	1.68	0.001
**Covariate 5**									
ADHD medication	4.02	0.72	< 0.001	4.11	0.61	< 0.001	5.33	2.29	0.020

Bold values indicates statistical significance

*p < 0.05.

*ADHD* attention-deficit/hyperactivity disorder, *ASD* autism spectrum disorder, *CBCL* the Child Behavior Checklist, *ABCL* Adult Behaviour Checklist.

## Results

### Comparisons of physical problems between ADHD and non-ADHD

In the total twin sample of all ages, participants with ADHD diagnosis had significantly more neurological problems (t = 2.55, p = 0.012) compared to those without ADHD (Table 2 [a]). However, when dividing the sample into children group and adult group, the differences of the frequency of neurological problems between ADHD and non-ADHD became non-significant in both groups. Still, there was a trend that children participants with ADHD had more neurological health issues than controls (t = 2.28, p = 0.024, larger than 0.0125, the adjusted value for statistical significance here). For infectious diseases, gastrointestinal problems, and immunological problems, there was no difference found between participants diagnosed with ADHD and non-ADHD twins. Comparisons within pairs of MZ and DZ twins discordant for ADHD diagnosis and dimensional ADHD symptoms revealed no difference of co-existing physical problems between ADHD twins and co-twins (Table 2 [b], [c]).

### The association between neurological comorbidity and ADHD diagnosis as well as symptoms of ADHD for the whole sample

Neurological conditions were significantly associated with the diagnosis of ADHD in children, even when controlling for the possible confounders (β = 0.38, p = 0.046, odds ratio = 1.46 per neurological problem [95% confidence interval = 1.01–2.12]), such as age, gender, a comorbid diagnosis of ASD, and level of IQ (Table 3 [a]). However, there was no significant association found in the all-age group and in the adult group. For dimensional ADHD symptoms, neurological problems were not associated with the scores of CBCL/ABCL-AP in the analyses of all groups with different ages after adjusting for ADHD medication (Table 3 [b]).

### Within-pair effect of physical comorbidity on ADHD symptoms for dimensionally discordant twin pairs

In the conditional logistic model, within-twin pair increases in GI problems were associated with decreases in CBCL-AP subscale scores in MZ children twins quantitatively differing for ADHD after adjusting for ADHD medication (β =  − 2.72, p = 0.001, Table 4), but not for all-age group of MZ twins and not for DZ twins. There was no significant within-pair association with ADHD symptoms found for infectious diseases, neurological problems, and immunological problems in all age groups of MZ and DZ twins.

**Table 4:** Associations between physical problems and ADHD symptoms in MZ and DZ twin pairs differing for ADHD symptoms (p value unadjusted).

	Outcome: ADHD symptoms (CBCL/ABCL-attention subscale)											
	Monozygotic twins						Dizygotic twins					
	All ages, n = 44 pairs			Children, n = 33 pairs			All ages, n = 61 pairs			Children, n = 44 pairs		
	β	s.e	p	β	s.e	p	β	s.e	p	β	s.e	p
**Exposure variable**												
Infectious diseases	− 0.27	0.24	0.257	− 0.32	0.25	0.197	− 1.29	0.97	0.183	− 0.37	0.94	0.690
**Covariate 1**												
Full-scale IQ	− 0.13	0.05	0.011	− 0.16	0.08	0.033	− 0.10	0.05	0.059	− 0.10	0.06	0.082
**Covariate 2**												
ASD	1.09	1.33	0.416	0.53	1.74	0.760	3.19	1.70	0.061	0.88	1.93	0.648
**Covariate 3**												
ADHD medication	2.94	1.46	0.044	1.42	0.65	0.029	4.75	1.50	0.001	6.57	1.20	< 0.001
**Covariate 4**												
Gender	–	–	–	–	–	–	− 2.08	1.16	0.074	− 1.75	1.11	0.116
**Exposure variable**												
Neurological Problems	− 0.20	0.73	0.786	0.00	0.78	0.995	− 0.96	1.23	0.436	0.25	1.27	0.842
**Covariate 1**												
Full-scale IQ	− 0.14	0.06	0.016	− 0.16	0.08	0.039	− 0.12	0.06	0.042	− 0.10	0.06	0.092
**Covariate 2**												
ASD	1.33	1.35	0.325	0.57	1.80	0.750	2.84	1.57	0.071	0.72	1.84	0.697
**Covariate 3**												
ADHD medication	3.11	1.71	0.070	1.41	1.31	0.280	4.69	1.61	0.004	6.81	1.25	< 0.001
**Covariate 4**												
Gender	–	–	–	–	–	–	− 1.96	1.22	0.110	− 1.91	1.18	0.105
**Exposure variable**												
Gastrointestinal problems	− 1.69	1.01	0.095	− 2.72	0.83	**0.001**	2.97	1.76	0.092	0.50	2.39	0.834
**Covariate 1**												
Full-scale IQ	− 0.11	0.05	0.040	− 0.16	0.07	0.035	− 0.09	0.05	0.070	− 0.10	0.06	0.069
**Covariate 2**												
ASD	1.25	1.19	0.294	0.26	1.52	0.867	2.55	1.64	0.121	0.69	1.79	0.700
**Covariate 3**												
ADHD medication	2.83	1.88	0.131	0.27	1.27	0.833	5.87	0.99	< 0.001	6.74	1.12	< 0.001
**Covariate 4**												
Gender	–	–	–	–	–	–	− 1.93	0.94	0.040	− 1.80	1.10	0.100
**Exposure variable**												
Immunological Problems	− 0.76	0.78	0.326	− 0.71	0.86	0.409	− 0.92	0.78	0.238	− 0.84	0.82	0.308
**Covariate 1**												
Full-scale IQ	− 0.12	0.06	0.046	− 0.15	0.08	0.063	− 0.10	0.05	0.034	− 0.10	0.05	0.060
**Covariate 2**												
ASD	1.16	1.38	0.400	0.59	1.81	0.745	3.34	1.71	0.050	1.27	1.97	0.519
**Covariate 3**												
ADHD medication	2.89	1.61	0.072	1.15	0.65	0.077	4.41	1.61	0.006	6.31	1.20	< 0.001
**Covariate 4**												
Gender	–	–	–	–	–	–	− 2.33	0.99	0.018	− 1.97	0.92	0.032

Bold value indicate statistical significance

*p < 0.0125.

With Bonferroni correction, the significance level of p values in this table is set at p < 0.0125.

*ADHD* attention-deficit/hyperactivity disorder, *ASD* autism spectrum disorder, *CBCL* the Child Behavior Checklist, *ABCL* Adult Behaviour Checklist.

## Discussion

This is the first study to investigate the association between co-existing physical problems and ADHD using a well characterized twin sample enriched for NDDs. In addition, we examined the role of physical health issues on the severity of ADHD symptoms in twins quantitatively differing for ADHD. Our results revealed that neurological problems among children were associated with the diagnosis of ADHD. However, for MZ twins with differing dimensional ADHD symptoms, GI problems showed protective within pair effects, even after adjusting for ADHD medications.

Our findings support that ADHD is a neurodevelopmental condition, in which the complex underpinning of altered brain development might not only affect multiple domains of cognitive and other behavioural function, but also increase the susceptibility of neurological health issues^45−47^. Moreover, childhood GI tract health could be considered as a non-shared environmental factor associated with severity of ADHD symptoms, which might exert influence via the interplay with a specific genetic background. More research is warranted to disentangle the mechanisms contributing to the overlap between ADHD and neurological complications, as well as the role of gut-brain axis on the phenotypic variation of ADHD^20^.

We examined the differences in frequency of physical problems between ADHD and non-ADHD individuals within our sample and the results varied for different physical systems. In line with the previous literature^22,48^, individuals with ADHD showed higher rates of neurological health issues, despite the fact that the result disappeared when data was analysed separately in children and adults. We did not observe differences between ADHD and non-ADHD in terms of infection history, co-existing GI problems, and comorbid immunological diseases, in contrast to the results of prior epidemiological studies and also meta-analyses which synthesized data of asthma, atopic diseases, and allergic diseases in ADHD^10,11,22,49−51^. Still, these discrepancies may predominantly reflect the relatively small effect sizes of the differences between ADHD and the general population in general, as well as the heterogeneity of ADHD individuals from aetiology to clinical profiles.

The reported odds ratios of neurological, immunological, infectious and digestive problems in ADHD compared to non-ADHD are probably low and less than 1.5^22,50^. Therefore, it is likely that large samples are required to detect such modest differences of physical comorbidity between individuals with and without ADHD. Moreover, although the prevalence of physical comorbidity in ADHD has been reported to be increased, there is still information lacking regarding whether and how much of these co-occurring physical problems account for the variation of ADHD symptoms^52^. Also, these physical comorbidities are only present in a minority of the ADHD population, rather than accompanying ADHD symptoms consistently. In view of the heterogeneity in aetiology and possible biological pathways of ADHD^14^, the mechanisms underlying the overlap of ADHD and physical health issues are likely to be various across individuals. Thus, the results of prevalence studies investigating physical health in ADHD could be more consistent in population-based samples and varying more among clinical samples or samples that do not exemplify the full spectrum of traits in the target population^10,11,49^. Finally, our sample included both children and adults. However, ADHD is a developmental condition with changing symptomatology over time, which might reflect the dynamic influences of genetics, surroundings, psychosocial factors, and the maturation progress of brain function^25,53−55^. Individuals with ADHD whose symptoms continue to meet the diagnostic criteria when they move into adulthood, only comprise about 50% of the childhood ADHD population^56^. Research revealed that adult ADHD, or the non-remitters, may have distinct risk gene variants^57^. In addition, the psychiatric comorbidity pattern of ADHD also changes throughout the lifespan, such as the increasing rates of personality disorders and bipolar disorders in adulthood^58^. Hence, adult ADHD might be a specific subgroup in terms of genetic basis and developmental trajectory, and could also exhibit different profiles of comorbid physical diseases^59^. For instance, the prevalence of obesity in adult ADHD was found to be more than twice as high as the one for childhood ADHD (28.2% vs 10.3%)^12^. More studies on physical comorbidity in adult ADHD are needed to further enhance our understanding of whether the association between ADHD and physical health issues persists into adulthood.

Our results showed that neurological problems are associated with ADHD diagnosis in childhood. Although alterations in attentional capacity can be secondary to frequent seizure attacks, headache episodes, and antiepileptic medication^60−64^, the found association might suggest that children with ADHD are more vulnerable to neurological health issues subsequently, as prospective associations found in previous studies indicate^7,46,65^. Mechanisms contributing to the higher comorbidity rate between ADHD and specific neurological problems have been proposed. From a biological perspective, predisposing genes^66,67^, disturbances of the norepinephrine and dopamine systems^47,68^, and altered brain functional networks^69^ have been postulated to be involved in the co-occurrence of ADHD, epilepsy, and migraine. The association between ADHD and headaches could be mediated by other psychiatric problems, such as sleep disorders and affective disorders^47,70^. Moreover, headache has been commonly reported as one of more frequent side effects of ADHD medications^71^.

Given the possible disabling consequences of neurological complications, it is imperative for practitioners working with ADHD to be attentive and to provide adequate management for the comorbid nervous system conditions in this population. Likewise, ADHD should also be screened for in children with neurological problems for early identification and intervention^18^. Regarding the association between neurological conditions and the severity of ADHD symptoms, we did not find a significant association. Since the individuals with ADHD in our sample were not drug-naïve, possible the treatment and side effects of ADHD medications could not be ruled out as a moderator influencing the outcomes. Further investigations with medication naïve subjects would be helpful to explore the impact of neurological health issues on ADHD symptoms.

Based on our analysis of within pair effects of physical comorbidity, GI system health could be considered a non-shared environmental factor to the severity of ADHD symptoms among monozygotic twin children. Among the digestive problems presented in the twins with less severe ADHD symptoms, lactose intolerance accounted for the majority of those (7/12, 58.3%). The others were irritable bowel syndrome, abdominal pain, intestinal polyps, diarrhea, and gastroenteritis. We speculate that those children with GI tract health issues were more likely to have diet adjustment to avoid food possibly inducing GI symptoms^72,73^. Their caregivers and relatives might also pay more attention to their diet preparation, including nutritional balance and elimination of food with artificial additives^74^. The diet alteration may change the gut microbiota^75^, which had been proposed to link with the potential pathophysiology of ADHD symptoms through vagus nerve, neuro-metabolites, and neuroinflammation pathways^20^. In addition, the association between possible dietary change and improvement of ADHD symptoms might reflect the effectiveness of diet intervention in previous double-blind placebo-controlled trials in children with ADHD^76^. However, our results did not demonstrate similar effects of existence of digestive problems among dizygotic twins, whose genetic makeup differs. This may indicate that the contribution of GI tract health to the variation of ADHD symptoms could be synergistic under certain circumstances, such as a genetic predisposition towards ADHD. This is consistent with the findings from trials of diet treatment, in that only a subgroup of children with ADHD responded to the administration of dietary change^76^. Therefore, it is recommended for future research to focus on the predictors of recognizing those children who would benefit from diet intervention, as well as the mechanisms underlying the association of diet, digestive problems, and ADHD symptoms. Still, other alternative explanations for the inverse association between GI problems and ADHD symptoms are needed to be considered. For instance, children’s gastrointestinal illness might increase parents’ tolerance for their children’s behavioral problems.

There are several limitations to this study that need to be addressed. First, although mainly selected from a population-based study^30,31^, some twins participating in RATSS were also recruited from via other sources and not sampled randomly, why the overall limited representativeness of our study sample and the generalizability of the results must be kept in mind regarding the frequency of physical problems. Moreover, our findings from a twin sample should be interpreted with caution when extending to singleton samples. Twin pregnancy has increased risk for perinatal morbidity^77^, which is associated with neurodevelopmental conditions and neurological complications^3,78^. Second, the sample size of adult ADHD in our study was limited, making results for association between physical comorbidity and ADHD among children and adults harder to compare. In addition, we did not have enough pairs of twins discordant for ADHD diagnosis to explore the within pair effect of physical problems on the clinical phenotypes of ADHD. Third, the CBCL/ABCL-AP is not a symptom scale derived from DSM-5, making analysis for categorical ADHD and dimensional ADHD symptom less comparable. Despite the validity of CBCL/ABCL-AP for identifying ADHD^79^, instruments designed for quantifying ADHD symptoms such as the Conners Rating Scale-Revised (CRS-R)^80^, the Swanson, Nolan, and Pelham Questionnaire (SNAP-IV)^81^, and adult ADHD self-report scale (ASRS)^82^ should also be considered in future studies to measure the core symptoms of ADHD more directly. Fourth, information on physical comorbidity and medical history of infectious diseases was reported by either parents of children with ADHD or adult participants with ADHD via questionnaires. Thus a risk of reporting and recall bias which might lead to an overestimation of the found associations cannot be ruled out. Fifth, the contribution of each physical problem to the association with ADHD symptoms may not be equal, which means the effect size of each problem in the association analysis could be varying. Therefore, the results of our study might be limited with the unweighted approach. Still, the grouping of physical problems might not be accurate either with regard to underlying mechanisms. Sixth, we were unable to confirm whether twins with more digestive problems compared to their co-twin were administered diet adjustments by their parents or not. Further investigations are needed to clarify the association between dietary change and the variation of ADHD symptoms among twins dimensionally differing for ADHD. Finally, our participants with ADHD were not free from treatment. Hence, our results could also be limited in view of the effects of ADHD medication or non-pharmacological interventions.

In conclusion, controlling for the contribution of complex genetics and other common confounders, our findings suggest that health conditions of digestive system are associated with ADHD symptom presentation among twins, and thus form a non-shared environmental factor for behavioural phenotypes in ADHD. Hence, our results support that the gut-brain axis might play some role in the underpinnings of ADHD symptoms, at least for a subgroup of individuals with certain genetic predisposition. In non-responders and those intolerant to ADHD medications, it could be of clinically valuable to identify those individuals with ADHD who might benefit from diet treatment, which has been believed to alter the gut microbiota composition^75^. In addition, we found that neurological problems are associated with ADHD diagnosis among children, in line with previous prevalence studies. Since the emergence of neurological conditions could be either prior to or subsequent to ADHD, it is recommended for clinicians to be aware of the higher rate of comorbidity, and to provide adequate assessment and intervention to improve both physical and psychosocial outcomes of children with ADHD and neurological health issues.

## References

[1] American Psychiatric Association (2013). Diagnostic and Statistical Manual of Mental Disorders.

[2] Faraone SV (2018). The pharmacology of amphetamine and methylphenidate: Relevance to the neurobiology of attention-deficit/hyperactivity disorder and other psychiatric comorbidities. Neurosci. Biobehav. Rev..

[3] Posner J, Polanczyk GV, Sonuga-Barke E (2020). Attention-deficit hyperactivity disorder. Lancet.

[4] Faraone SV, Larsson H (2019). Genetics of attention deficit hyperactivity disorder. Mol. Psychiatry..

[5] Banerjee TD, Middleton F, Faraone SV (2007). Environmental risk factors for attention-deficit hyperactivity disorder. Acta. Paediatr..

[6] Stevenson J (2014). Research Review: The role of diet in the treatment of attention-deficit/hyperactivity disorder—An appraisal of the evidence on efficacy and recommendations on the design of future studies. J. Child. Psychol. Psychiatry..

[7] Hesdorffer DC (2004). ADHD as a risk factor for incident unprovoked seizures and epilepsyin children. Arch. Gen. Psychiatry..

[8] Aaberg KM (2016). Comorbidity and childhood epilepsy: A nationwide registry study. Pediatrics.

[9] Salem H (2018). ADHD is associated with migraine: A systematic review and meta-analysis. Eur. Child. Adolesc. Psychiatry..

[10] Schans JV, Çiçek R, de Vries TW, Hak E, Hoekstra PJ (2017). Association of atopic diseases and attention-deficit/hyperactivity disorder: A systematic review and meta-analyses. Neurosci. Biobehav. Rev..

[11] Cortese S (2018). Association between attention deficit hyperactivity disorder and asthma: A systematic review and meta-analysis and a Swedish population-based study. Lancet. Psychiatry..

[12] Cortese S (2016). Association between ADHD and obesity: A systematic review and meta-analysis. Am. J. Psychiatry..

[13] Prehn-Kristensen A (2018). Reduced microbiome alpha diversity in young patients with ADHD. PLoS ONE.

[14] Luo Y, Weibman D, Halperin JM, Li X (2019). A review of heterogeneity in attention deficit/hyperactivity disorder (ADHD). Front. Hum. Neurosci..

[15] Austin JK (2001). Behavior problems in children before first recognized seizures. Pediatrics.

[16] Vega C (2010). Differentiation of attention-related problems in childhood absence epilepsy. Epilepsy. Behav..

[17] McCusker CG, Kennedy PJ, Anderson J, Hicks EM, Hanrahan D (2002). Adjustment in children with intractable epilepsy: Importance of seizure duration and family factors. Dev. Med. Child. Neurol..

[18] Auvin S (2018). Systematic review of the screening, diagnosis, and management of ADHD in children with epilepsy. Consensus paper of the Task Force on Comorbidities of the ILAE Pediatric Commission. Epilepsia..

[19] Dunn GA, Nigg JT, Sullivan EL (2019). Neuroinflammation as a risk factor for attention deficit hyperactivity disorder. Pharmacol. Biochem. Behav..

[20] Dam SA (2019). The role of the gut-brain axis in attention-deficit/hyperactivity disorder. Gastroenterol. Clin. N. Am..

[21] Heijtz RD (2011). Normal gut microbiota modulates brain development and behavior. Proc. Natl. Acad. Sci. USA.

[22] Kline-Simon AH, Weisner C, Sterling S (2016). Point prevalence of co-occurring behavioral health conditions and associated chronic disease burden among adolescents. J. Am. Acad. Child. Adolesc. Psychiatry..

[23] Niederhofer H (2011). Association of attention-deficit/hyperactivity disorder and celiac disease: A brief report. Prim. Care. Companion. CNS. Disord..

[24] Demontis D (2019). Discovery of the first genome-wide significant risk loci for attention deficit/hyperactivity disorder. Nat. Genet..

[25] Chang Z, Lichtenstein P, Asherson PJ, Larsson H (2013). Developmental twin study of attention problems: high heritabilities throughout development. JAMA. Psychiatry..

[26] Hultman CM (2007). Birth weight and attention-deficit/hyperactivity symptoms in childhood and early adolescence: A prospective Swedish twin study. J. Am. Acad. Child. Adolesc. Psychiatry..

[27] Lim KX (2018). The role of birth weight on the causal pathway to child and adolescent ADHD symptomatology: A population-based twin differences longitudinal design. J. Child. Psychol. Psychiatry..

[28] Tye C, Runicles AK, Whitehouse A, Alvares GA (2019). Characterizing the interplay between autism spectrum disorder and comorbid medical conditions: An integrative review. Front. Psychiatry..

[29] Sappok T, Diefenbacher A, Winterholler M (2019). The medical care of people with intellectual disability. Dtsch. Arztebl. Int..

[30] Bölte S (2014). The roots of autism and ADHD twin study in Sweden (RATSS). Twin. Res. Hum. Genet..

[31] Anckarsäter H (2011). The child and adolescent twin study in Sweden (CATSS). Twin. Res. Hum. Genet..

[32] Stamouli S (2018). Copy number variation analysis of 100 twin pairs enriched for neurodevelopmental disorders. Twin. Res. Hum. Genet..

[33] Willfors C (2017). Medical history of discordant twins and environmental etiologies of autism. Transl. Psychiatry..

[34] Kaufman J (1997). Schedule for affective disorders and schizophrenia for school-age children-present and lifetime version (K-SADS-PL): Initial reliability and validity data. J. Am. Acad. Child. Adolesc. Psychiatry..

[35] Kooij JJS, Francken MH (2010). Diagnostic Interview for ADHD in Adults (DIVA 2.0).

[36] Rutter M, Le Couteur A, Lord C (2003). Autism Diagnostic Interview Revised (ADI-R).

[37] Lord C (2010). Autism Diagnostic Observation Schedule, Second Edition (ADOS-2) Manual (Part I).

[38] Wechsler D (2003). WISC-IV Technical and Interpretive Manual.

[39] Wechsler D (2008). WAIS-IV Technical and Interpretative Manual.

[40] Achenbach TM, Rescorla LA (2001). Manual for the ASEBA School-Age Forms & Profiles.

[41] Achenbach, T. M. *Manual for the ASEBA Adult Forms & Profiles: For Ages 18–59 : Adult Self-Report and Adult Behavior Checklist*. (Aseba, 2003).

[42] Pan P-Y, Tammimies K, Bölte S (2020). The association between somatic health, autism spectrum disorder, and autistic traits. Behav. Genet..

[43] Zetterqvist J, Vansteelandt S, Pawitan Y, Sjölander A (2016). Doubly robust methods for handling confounding by cluster. Biostatistics..

[44] Morey, R.D. *et al.* Computation of Bayes factors for common designs. https://cran.r-project.org/web/packages/BayesFactor/BayesFactor.pdf (2018).

[45] Reiss AL (2009). Childhood developmental disorders: An academic and clinical convergence point for psychiatry, neurology, psychology and pediatrics. J. Child. Psychol. Psychiatry..

[46] Hermann B (2007). The frequency, complications and aetiology of ADHD in new onset paediatric epilepsy. Brain.

[47] Parisi P (2014). Headache and attention deficit and hyperactivity disorder in children: Common condition with complex relation and disabling consequences. Epilepsy. Behav..

[48] Park KJ, Lee JS, Kim HW (2017). Medical and psychiatric comorbidities in Korean children and adolescents with attention-deficit/hyperactivity disorder. Psychiatry. Investig..

[49] Miyazaki C (2017). Allergic diseases in children with attention deficit hyperactivity disorder: A systematic review and meta-analysis. BMC. Psychiatry..

[50] Jameson ND (2016). Medical comorbidity of attention-deficit/hyperactivity disorder in US adolescents. J. Child. Neurol..

[51] Akmatov MK, Ermakova T, Bätzing J (2003). Psychiatric and nonpsychiatric comorbidities among children with ADHD: An exploratory analysis of nationwide claims data in Germany. J. Atten. Disord..

[52] Holtmann M, Becker K, Kentner-Figura B, Schmidt MH (2003). Increased frequency of rolandic spikes in ADHD children. Epilepsia..

[53] Willcutt EG (2012). Validity of DSM-IV attention deficit/hyperactivity disorder symptom dimensions and subtypes. J. Abnorm. Psychol..

[54] Lahey BB, Pelham WE, Loney J, Lee SS, Willcutt E (2005). Instability of the DSM-IV subtypes of ADHD from preschool through elementary school. Arch. Gen. Psychiatry..

[55] Pingault JB (2015). Genetic and environmental influences on the developmental course of attention-deficit/hyperactivity disorder symptoms from childhood to adolescence. JAMA. Psychiatry..

[56] Sudre G, Mangalmurti A, Shaw P (2018). Growing out of attention deficit hyperactivity disorder: Insights from the 'remitted' brain. Neurosci. Biobehav. Rev..

[57] Palladino VS, McNeill R, Reif A, Kittel-Schneider S (2019). Genetic risk factors and gene-environment interactions in adult and childhood attention-deficit/hyperactivity disorder. Psychiatr. Genet..

[58] Franke B (2018). Live fast, die young? A review on the developmental trajectories of ADHD across the lifespan. Eur. Neuropsychopharmacol..

[59] Instanes JT, Klungsøyr K, Halmøy A, Fasmer OB, Haavik J (2018). Adult ADHD and comorbid somatic disease: A systematic literature review. J. Atten. Disord..

[60] Sánchez-Carpintero R, Neville BG (2003). Attentional ability in children with epilepsy. Epilepsia..

[61] Kwan P, Brodie MJ (2001). Neuropsychological effects of epilepsy and antiepileptic drugs. Lancet.

[62] Kavros PM (2008). Attention impairment in rolandic epilepsy: Systematic review. Epilepsia..

[63] Villa TR (2009). Visual attention in children with migraine: A controlled comparative study. Cephalalgia.

[64] Riva D (2012). Attention in children and adolescents with headache. Headache..

[65] Genizi J (2013). Primary headaches, attention deficit disorder and learning disabilities in children and adolescents. J. Headache. Pain..

[66] Kutuk MO (2018). Migraine and associated comorbidities are three times more frequent in children with ADHD and their mothers. Brain. Dev..

[67] Brikell I (2018). Familial liability to epilepsy and attention-deficit/hyperactivity disorder: A nationwide cohort study. Biol. Psychiatry..

[68] Pineda E (2014). Behavioral impairments in rats with chronic epilepsy suggest comorbidity between epilepsy and attention deficit/hyperactivity disorder. Epilepsy. Behav..

[69] Yoong M (2015). Quantifying the deficit-imaging neurobehavioural impairment in childhood epilepsy. Quant. Imaging. Med. Surg..

[70] Minen MT (2016). Migraine and its psychiatric comorbidities. J. Neurol. Neurosurg. Psychiatry..

[71] Ching C, Eslick GD, Poulton AS (2019). Evaluation of methylphenidate safety and maximum-dose titration rationale in attention-deficit/hyperactivity disorder: A meta-analysis. JAMA. Pediatr..

[72] Korterink J, Devanarayana NM, Rajindrajith S, Vlieger A, Benninga MA (2015). Childhood functional abdominal pain: Mechanisms and management. Nat. Rev. Gastroenterol. Hepatol..

[73] Misselwitz B, Butter M, Verbeke K, Fox MR (2019). Update on lactose malabsorption and intolerance: Pathogenesis, diagnosis and clinical management. Gut.

[74] Howard AL (2011). ADHD is associated with a “Western” dietary pattern in adolescents. J. Atten. Disord..

[75] David LA (2014). Diet rapidly and reproducibly alters the human gut microbiome. Nature.

[76] Pelsser LM, Frankena K, Toorman J, Rodrigues Pereira R (2017). Diet and ADHD, reviewing the evidence: A systematic review of meta-analyses of double-blind placebo-controlled trials evaluating the efficacy of diet interventions on the behavior of children with ADHD. PLoS ONE.

[77] Santana DS, Surita FG, Cecatti JG (2018). Multiple pregnancy: Epidemiology and association with maternal and perinatal morbidity. Rev. Bras. Ginecol. Obstet..

[78] Sadowska M, Sarecka-Hujar B, Kopyta I (2020). Cerebral palsy: Current opinions on definition, epidemiology, risk factors, classification and treatment options. Neuropsychiatr. Dis. Treat..

[79] Chang LY, Wang MY, Tsai PS (2016). Diagnostic accuracy of rating scales for attention-deficit/hyperactivity disorder: A meta-analysis. Pediatrics.

[80] Conners CK (1997). Conners’ Rating Scales-Revised Technical Manual.

[81] Swanson JM (2001). Clinical relevance of the primary findings of the MTA: Success rates based on severity of ADHD and ODD symptoms at the end of treatment. J. Am. Acad. Child. Adolesc. Psychiatry..

[82] Kessler RC (2005). The World Health Organization Adult ADHD Self-Report Scale (ASRS): A short screening scale for use in the general population. Psychol. Med..

